# The development of new biomarkers of spermatozoa quality in cattle

**DOI:** 10.3389/fvets.2023.1258295

**Published:** 2023-10-12

**Authors:** Lindsey Fallon, Edgar Diaz-Miranda, Lauren Hamilton, Peter Sutovsky, Michal Zigo, Thomas E. Spencer, M. Sofia Ortega

**Affiliations:** ^1^Division of Animal Sciences, University of Missouri, Columbia, MO, United States; ^2^Department of Veterinary, Universidade Federal de Viçosa, Viçosa, MG, Brazil; ^3^Department of Obstetrics, University of Missouri, Columbia, MO, United States; ^4^Department of Gynecology & Women’s Health, University of Missouri, Columbia, MO, United States

**Keywords:** spermatozoa morphology, sire fertility, aggresomes, semen processing, flow cytometry

## Abstract

There is a current need for new biomarkers of spermatozoa quality, that consistently and correctly identify spermatozoa that will successfully contribute to subsequent embryo development. This could improve the standardization of semen analysis, decrease early embryo mortality, and use these biomarkers as a selection tool before servicing females. This study utilized imaging techniques to identify potential biomarkers of sperm quality, using sires previously classified as high (*n* = 4) or low (*n* = 4) performing at producing blastocysts *in vitro.* Spermatozoa were assessed before and following a gradient purification protocol, to understand how populations of cells are impacted by such protocols and may differ between *in vivo* and *in vitro* use. Pre-gradient samples from low-performing sires had an increased incidence of DNA damage, although post-gradient samples from high-performing sires were found to have an increased incidence of DNA damage. When evaluating morphology via fluorescent microscopy, the most prevalent defects in pre-gradient samples from high-performing sires were tail defects, which are successfully removed during purification processing. The most prevalent defects in pre-gradient samples from low-performing sires were aggresome defects located in the head, which would be brought into an oocyte upon fertilization and may impair embryo development. Image-based flow cytometry (IBFC) was employed to quantify defect prevalence to evaluate a greater sample size decreasing the variability that exists in manual assessments. Using IBFC, aggresome defects were again identified in the heads of spermatozoa from low-performing sires. Post-gradient samples from low-performing sires had a significantly greater (*p* < 0.05) incidence of aggresome defects than post-gradient samples from high-performing sires. Additionally, IBFC was used to evaluate spermatozoa viability following gradient purification. Distinct populations of sperm cells were identified. High-performing sires had more spermatozoa in the population deemed most viable than low-performing sires. This study demonstrated that spermatozoa defects vary in populations before and following gradient purification, indicating that it may be beneficial to separately evaluate semen for *in vivo* and *in vitro* use. Furthermore, a prevalent defect in low-performing sires that could explain a discrepancy between successful fertilization and embryo development was identified. Therefore, elucidating a malfunction regulated by sire, that could potentially affect early embryo development.

## Introduction

There is currently an outstanding need for the identification of reliable biomarkers of spermatozoa quality in cattle. Until recent years, sperm evaluations during semen processing only included manual assessment of crude morphological features and motility. Although microscopy-based assessments provide valuable insight into the spermatozoa’s ability to reach and fertilize an oocyte, they cannot differentiate which spermatozoa will contribute to a viable embryo past the penetration of the vitelline membrane. Additionally, manual semen analysis is subjective and lacks standardization across facilities and technicians (1).

Early embryo mortality is a primary factor that contributes to economic loss in the dairy cattle industry, with up to 50% of pregnancy loss occurring within the first 7 days following fertilization (2). The cost of a single pregnancy loss has been estimated at approximately $555 in the United States but can vary greatly by country of production, and has been estimated to exceed 5,000 pesos in Mexico (3, 4). More accurate identification of sire fertility could help to ameliorate this issue, as a sire exhibits a great impact on pregnancy success during fertilization, preimplantation embryo development, conceptus elongation, and placentation (5, 6). No single bioassay can accurately classify sire fertility, although, a combination of multiple evaluations provides a correlation to a sire’s ability to successfully produce a viable pregnancy (7, 8).

In recent years, the use of techniques such as computer-assisted sperm analysis (CASA) and flow cytometry have allowed for more objective quantification of semen quality (8). The CASA system incorporates videos of sperm microscopy to train computer software programs to evaluate sperm concentration, motility, and morphology (1). Although this system reduces technician error in manual evaluation, studies with human samples have shown that CASA accuracy decreases with decreased sperm concentrations and therefore fewer cells per field of view (1, 9). Previous literature has described the implementation of flow cytometric analyses with fluorescently labeled sperm cells in human fertility clinics as a way to alleviate the variation in accuracy due to concentration (1, 10, 11). Using this technology, tens of thousands of sperm cells per sample can be accurately analyzed for one or more features of interest in just minutes, significantly increasing the sample size and decreasing evaluation variability (11). Flow cytometric sperm analysis is being further elevated by the implementation of machine learning algorithms, used with both fluorescent probes and label-free analyses, in conventional or image-based flow cytometry (12).

One important marker of sperm quality in live samples is acrosomal integrity, which is commonly assessed using fluorescently conjugated peanut (*Arachis hypogaea*) agglutinin (PNA), a lectin that binds to sugar moieties specifically present on the outer acrosomal membrane (13–15). Recently, the distribution of zinc (Zn^2+^) ion efflux has been used to monitor the progress of sperm capacitation in live samples and has been associated with varying degrees of male fertility (13). This correlation with fertility status may be due to zinc’s regulation of the HVCN1 proton channels and Catsper calcium channels, which are largely responsible for the sperm motility hyperactivation that is achieved simultaneously with sperm capacitation (13, 16). Additionally, subfertile and infertile bull spermatozoa have been found to exhibit increased ubiquitination which may be contributed by the ubiquitin-proteasome system (UPS) in conjunction with protein aggregation during spermatogenesis in the testis and sperm maturation in the epididymis (15, 17). Furthermore, increased ubiquitination of sperm cells has also been positively correlated with DNA damage (15, 18). The proportion of spermatozoa that exhibit negative fertility biomarkers can vary widely between ejaculates. However, through sperm processing techniques such as gradient purification, which is commonly used in preparation for *in vitro* fertilization (IVF), many of these cells can be removed from the sample. Therefore, it is beneficial to evaluate spermatozoa both before, and following processing techniques to gain a better understanding of the differences between the starting sperm population and the purified sperm population and how well defective cells can be removed from the sample (19).

The use of effective biomarkers independent of sire conception rate (SCR) could prove to be most beneficial to the industry due to SCR being determined by confirmed pregnancies at day 70–75 of gestation (20). SCR is defined as the probability that a given unit of semen from a specific bull will result in a viable pregnancy compared to an average bull, and is assigned only after AI 300 services have occurred within a 4-year period, which prevents its use for younger sires (21). A positive SCR value indicates increased fertility and a negative value indicates decreased fertility. Therefore, the identification of biomarkers independent of SCR would also allow the biomarkers to be used as a young sire selection tool prior to servicing females.

Previous studies in our laboratory have shown that sires vary in their ability to produce embryos in an *in vitro* culture system, independent of their SCR (22). Sires that were either high or low-performing at producing embryos, with varying SCR values, were then selected for subsequent experiments to elucidate mechanisms contributing to this variation. There was no difference observed between groups of sires in their ability to effectively fertilize oocytes and reach the pronuclear stage, although there were differences in developmental competence to the blastocyst stage. At the 2–6 cell stage of development, embryos produced by low-performing sires exhibited an increase in both reactive oxygen species (ROS) production and autophagic activity compared to embryos produced by high-performing sires—indicating that these embryos begin development under increased cellular stress (23).

These data makes the evaluation of spermatozoa from high and low-performing sires an ideal model to identify new biomarkers of *in vitro* sire fertility, independent of their SCR. Therefore, the current study sought to investigate if spermatozoa from sires with differing embryo production capacities have identifiable differences that could be exploited for biomarker development. By utilizing this model, we aim to identify new characteristics associated with *in vitro* sperm fertility in cattle and elucidate new paternal contribution mechanisms driving variation in embryo development.

## Materials and methods

### Embryo production

All media for oocyte collection and maturation, as well as *in vitro* embryo production, was prepared as previously described (6, 24). Briefly, cumulus-oocyte complexes (COCs) were collected from abattoir-derived ovaries. COCs with at least three layers of compact cumulus cells and homogeneous cytoplasm were selected and placed in an oocyte maturation medium warmed to 38.5°C and equilibrated with air containing 5% (v/v) CO_2_. COCs, in groups of 50, were left to mature for approximately 22 h before fertilization. Semen straws used in all experiments were gifted by Select Sires Inc. (Great Plains, OH, United States) and were processed in the same commercial house. Semen was collected from Holstein Sires at 28 months of age on average. Four high-performing sires and four low-performing sires were selected for the experiments of this study based on previous classification as having high or low capacity to produce embryos *in vitro* (22). Sperm was prepared for fertilization as previously described (6, 24) and diluted in fertilization medium (IVF-TALP) to a final concentration of 1 × 10^6^/mL in the fertilization plate. Mature oocytes and sperm were incubated together for 18–20 h at 38.5°C with air containing 5% (v/v) CO_2_, following which, cumulus cells were removed, and putative zygotes were placed in synthetic oviductal fluid (SOF-BEII) culture medium in a controlled environment [38.5°C with a humidified atmosphere of 5% (v/v) CO₂, 5% (v/v) O₂, and 90% (v/v) N₂]. Cleavage rates were assessed on the third day of embryo culture [66–72 h post insemination (HPI)] and blastocyst rates were assessed in the morning on the eighth day of embryo culture (186–192 HPI). Developmental data were collected throughout 47 embryo production rounds and analyzed using a generalized linear mixed model in the Statistical Analysis System version 9.4 (SAS Institute Inc., Cary, NC, United States). Data are presented as least squares means (LS means) ± standard error mean (SEM). Differences in means were identified using the pdiff option of LSMEANS.

### Semen preparation for analysis

Semen straws from the same sires used for embryo development were used for spermatozoa analyses. All semen straws were thawed in a water bath at 37°C for 40–45 s. Concomitantly, for samples that underwent gradient purification, 600 μL of 50% density upper layer gradient was gently pipetted onto 600 μL of 90% density lower layer gradient (Isolate™, Irvine Scientific), in a 1.5 mL microcentrifuge tube. Semen was extruded from the straw into the gradient tube and centrifuged at 700 × *g* for 5 min. The sperm pellet (100 μL) was removed from the bottom of the tube, placed into an Eppendorf tube containing 1 mL of warm Hepes-TALP medium (6), and centrifuged again at 700 × *g* for 3 min. This wash step was then repeated once more, for a total of two washes in Hepes-TALP per sample. For samples that did not undergo gradient purification, semen was extruded from the straw directly into 37°C warm Hepes-TALP and washed twice as described above.

### DNA damage evaluation

DNA integrity was assessed using the *In Situ* Cell Death Detection Kit (Roche Diagnostics, Catalog n 11684809910). Briefly, fixed sperm cells were placed into phosphate-buffered saline (PBS) on poly-L-lysine coated coverslips to adhere for 5 min. Cells were subsequently permeabilized for 40 min at room temperature (RT) by using PBS with 0.01% Triton X-100 (PBST). Following permeabilization, cells were incubated in the manufacturer-provided staining solution for 1 h at 37°C in a humidity chamber shielded from light. Cells were then counterstained with 4′,6-diamidino-2-phenylindole (DAPI, 1:200) for 15 min at RT before being washed for 5 min in PBS. Lastly, coverslips were mounted onto glass slides using Vectashield Mounting media (Vector Laboratories, Burlingame, CA, United States) and sealed with clear nail polish. A minimum of 200 cells were analyzed per sample at 40X magnification and images were recorded with a Nikon Eclipse 800 microscope (Nikon Instruments, Melville, NY, United States) equipped with a Retiga QI-R6 camera (Teledyne QImaging, Surrey, BC, Canada) operated by MetaMorph 7.10.2.240. software (Molecular Devices, San Jose, CA, United States). For all bulls, DNA damage assessments were conducted on both the overall sperm population (pre-gradient) and the gradient-separated pellet fraction (post-gradient) and were defined as having DNA damage if positive TUNEL labeling was observed. Data were analyzed with a binomial logistic regression model, using the GLIMMIX procedure of SAS. Data are presented as least squares means (LS means) ± standard error mean (SEM). Differences in means were identified using the pdiff option of LSMEANS.

### Morphology assessment via fluorescent microscopy

Fixed spermatozoa were placed into KMT buffer (100 mM KCl, 2 mM MgCl_2_, 10 mM Tris–HCl, 5 mM EGTA, pH 7) on poly-L-lysine coated coverslips to adhere for 20 min. Cells were then incubated in a staining solution for 30 min at RT shielded from light that contained fluorescent probes that label aggregated proteins (Proteostat Aggresome Detection Kit, ENZ-51035-K100, 1:2,000), the acrosome (PNA-FITC, 1:200), and DNA (DAPI, 1:200), diluted in PBST. Coverslips were subsequently washed twice, for 5 min each with PBS before they were mounted onto glass coverslips using Vectashield Mounting Media and sealed using clear nail polish. Approximately 110 cells per sample were imaged using differential interference contrast and epifluorescence at 100x magnification. Images were recorded with a Nikon Eclipse 800 microscope (Nikon Instruments, Melville, NY, United States) equipped with a Retiga QI-R6 camera (Teledyne QImaging, Surrey, BC, Canada) operated by MetaMorph 7.10.2.240. software (Molecular Devices, San Jose, CA, United States). The following morphological regions were analyzed for the presence of defects: acrosome (disrupted, ruffled, or knobbed acrosome), nuclear/head (nuclear crest, nuclear vacuoles, tapered head, microcephalic head, macrocephalic head, pyriform head, detached head, diadem, and multiple heads), tail midpiece (mitochondrial sheath disruption, midpiece reflex, stump-tailed, broken tails, and fracture), and tail principal piece (strongly coiled tail, principle piece reflex, abaxial tail, multiple tails, broken tail, and fracture); and the overall percentage of cells affected by each morphological defect was recorded. For all bulls, morphological assessments were conducted on both the overall sperm population (pre-gradient) and the gradient-separated pellet fraction (post-gradient) used for IVF. Data were analyzed with a binomial logistic regression model, using the GLIMMIX procedure in SAS, data are presented as least squares means (LS means) ± standard error mean (SEM). Differences in means were identified using the pdiff option of LSMEANS.

### Image-based flow cytometry with fixed samples

Following semen preparation, spermatozoa were fixed in 2% formaldehyde for 20 min before being subsequently incubated for 30 min in the dark at RT in a staining solution containing an aggresome probe (Proteostat Aggresome Detection Kit, ENZ-51035-K100, 1:10,000), PNA-FITC (1:2,500) and the nuclear stain Hoescht 33,342 (1,1,000) diluted in PBST. Lectin PNA was used to validate that permeabilization was achieved, such that the aggresome probe could target intracellular protein aggregates. Fixation was necessary for this analysis due to using the Proteostat Aggresome Detection Kit. After staining, samples were spun down using centrifugation (700 × *g*, 5 min) to remove the staining solution and resuspended in PBS for analysis. Samples were then analyzed with an Amnis FlowSight imaging flow cytometer (AMNIS Luminex Corporation, Austin, TX, United States) fitted with a 20X microscope objective (numerical aperture of 0.5) with an imaging rate of up to 2,000 events/s. The sheath fluid was PBS, free of Ca^2+^ and Mg^2+^. The flow-core size was 10 μm diameter and the speed was 66 mm/s, respectively. Raw images were acquired using INSPIRE® software (AMNIS Luminex Corporation). The camera was set to 1.0 μm per pixel of the charged-coupled device. The image display dimension for the field of view was 60 and 8 μm depth of field. Samples were analyzed using three lasers simultaneously: a 405-nm line with an intensity set to 10 mW; a 488-nm line with an intensity set to 30 mW; and a 785-nm line (side scatter) with an intensity set to 50 mW. A total of 18,000 to 23,000 events were evaluated by sire. Data analysis of the raw images was performed using the IDEAS® software (Version 6.3.23.0; AMNIS Luminex Corporation), where the electronic images were compensated for channel crossover by using single-color controls that were merged to generate a multi-color matrix. The compensation matrix file was then applied to a raw-image file (.rif), to create a color-compensated image file (.cif). A focused spermatozoa population was created by gating cells using Gradient RMS for the bright field channel. Single-cell events were gated by combining the Area × Aspect Ratio scatter plot of the brightfield, from the first step, with that of the DAPI channel. A single cell population gate was used for the histogram display of mean pixel intensities by frequency for the following channels: channels 1 and 9—brightfield, channel 2—green fluorescence (FITC, 505–560 nm) to capture acrosome labeling, channel 3—orange fluorescence (544–570 nm) to capture aggresome labeling, channel 6 (SSC), and channel 7—blue fluorescence (DAPI, 435–505 nm) to capture nuclear labeling. Intensity histograms for the individual channels were then used to gate sub-populations with varying intensity levels and visual conformations. A constant fluorescence intensity region from 683 to 10,625 was used as a gate to quantify the aggresome content (sperm head only and total) in all analyses. The percentage of spermatozoa that fell within the fluorescence intensity region of 683–10,625 for each evaluation were analyzed using a generalized linear mixed model in SAS and are expressed as least squares means (LS means) ± standard error mean (SEM).

### Image-based flow cytometry with live samples

Following semen preparation, approximately 5 million gradient purified spermatozoa were resuspended in HEPES-buffered Tyrode lactate medium supplemented with polyvinyl alcohol (TL-HEPES-PVA), containing 10 mM sodium lactate, 5.2 mM sodium pyruvate, 11 mM D-glucose, 0.5 mM MgCl_2_, and 0.01% (w/v) polyvinyl alcohol (PVA), and void of Ca^2+^ and HCO_3_^−^ ions; pH = 7.2, *t* = 37°C. The following probe combination was added to the sperm suspension to the final concentrations FluoZin-3, AM (1 μM), H33342 (18 μM), Propidium Iodide (PI, 1 μg∙mL^−1^), and CellROX™ Deep Red (10 μM). Sperm samples were left to incubate for 40 min at 37°C in the dark.

The fluorescently labeled samples were measured with the same Amnis FlowSight Imaging Flow Cytometer used in the prior evaluation of this study (AMNIS Luminex Corporation, Austin, TX, United States) and as described previously (13, 25). To produce the highest resolution, the camera setting was at 1.0 μm per pixel of the charge-coupled device. Samples were analyzed using four lasers concomitantly: a 405-nm line (20 mW), 488-nm line (60 mW), 642-nm line (75 mW), a 785-nm line (70 mW, side scatter), and two LEDs (32.57 and 19.30 mW respectively). Signals were observed in the following channels: channels 1 and 9—brightfield, channel 2—green fluorescence (FITC, 505–560 nm) to capture zinc labeling, channel 6 (SSC), channel 7—blue fluorescence (DAPI, 435–505 nm) to capture nuclear labeling, and channel 11—infrared fluorescence (AF647, DeepRed; 642–745 nm) to capture ROS labeling. A total of 18,000–23,000 events were evaluated by sire, and data were analyzed using IDEAS® software (Version 6.3.23.0; AMNIS Luminex Corporation, Austin, TX, United States). A focused, single-cell population gate with anteriorly/posteriorly oriented spermatozoa (13) was used for the histogram display of mean pixel intensities by frequency for collected channels. This gating technique allowed for uniformity in the orientation of spermatozoa that were analyzed in subsequent analyses. Intensity histograms of individual channels were then used for drawing regions of subpopulations with varying intensity levels and visual confirmation. The intensity of nuclear labeling was used for histogram normalization among samples. This gating technique allowed for the isolation of spermatozoa into populations based on if they were positive for the trait of interest (zinc, PI, ROS). The percentage of spermatozoa within each population were analyzed using a generalized linear mixed model in SAS and is expressed as least squares means (LS means) ± standard error mean (SEM). Differences in means were identified using the pdiff option of LSMEANS.

## Results

### Embryo development

**Figure 1:**
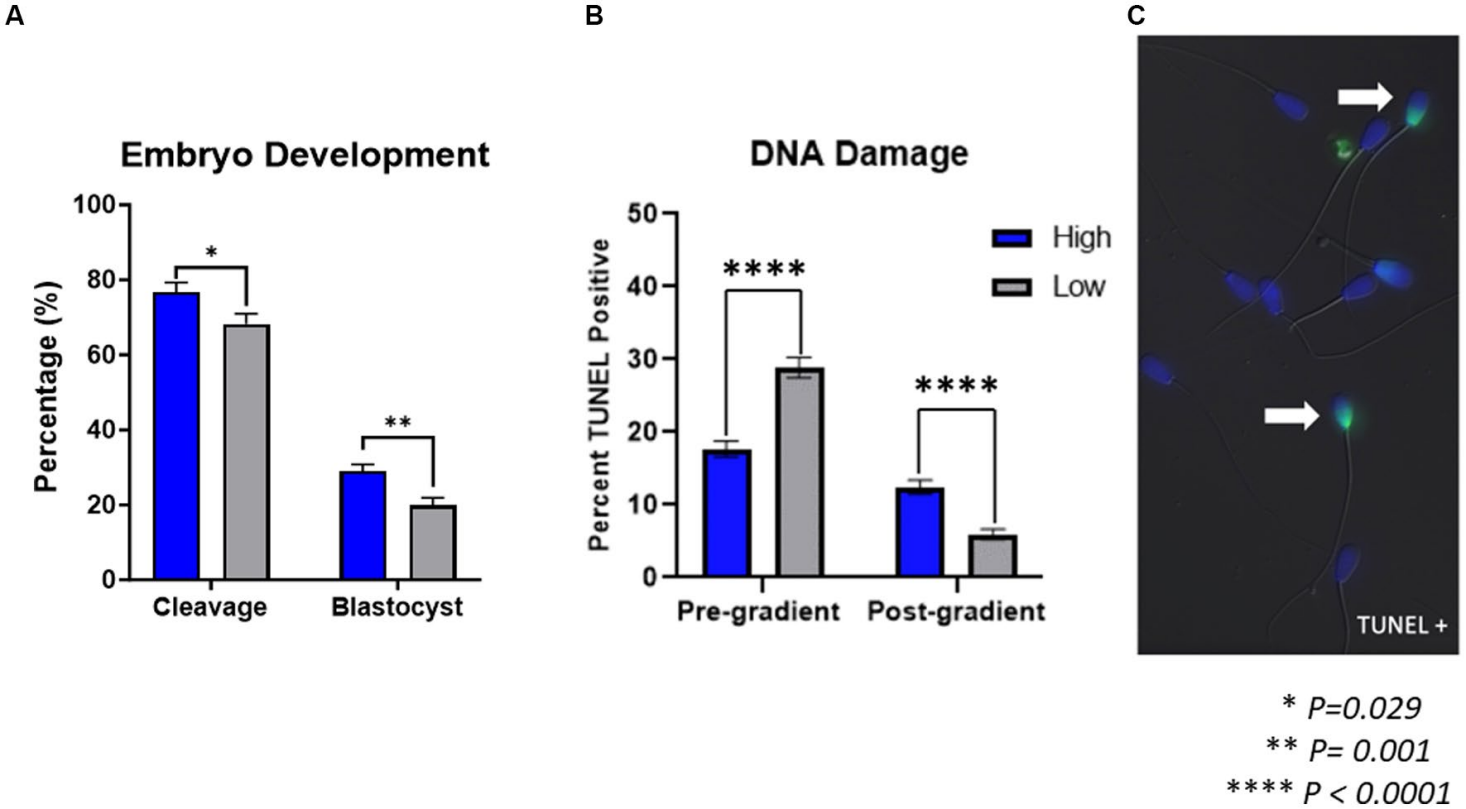
Embryo development and sperm DNA damage. **(A)** Embryos produced by high-performing sires had significantly higher cleavage rates (*p* = 0.029) and blastocyst rates (*p* = 0.001) than embryos produced by low-performing sires (*n* = 47 *in vitro* embryo production rounds). **(B)** In samples prior to gradient purification, termed pre-gradient samples, there was an increase in DNA damage present (*p* < 0.0001) in spermatozoa from low-performing sires compared to high-performing. Inversely, in samples following gradient purification, termed post-gradient samples, there was an increase in DNA damage present (*p* < 0.0001) in high-performing sires compared to low-performing (*n* = ~1,000 cells per classification). **(C)** Representative image of TUNEL positive spermatozoa.

Cleavage and blastocyst rates are shown in Figure 1A. Cleavage rates from embryos produced by high-performing sires (76.73 ± 2.57%) were significantly higher (*p* = 0.029) than cleavage rates from low-performing sires (68.25 ± 2.74%). Similarly, blastocyst rates from embryos produced by high-performing sires (29.04 ± 1.78%) were significantly higher (*p* = 0.001) than blastocyst rates from low-performing sires (19.94 ± 1.90%).

### DNA damage

The DNA damage present in spermatozoa from either high or low-performing sires is shown in Figure 1. The percent of TUNEL-positive sperm cells, indicating DNA damage, in samples prior to gradient purification was significantly increased (*p* < 0.0001) in samples from low-performing sires (28.85% ± 1.37) compared to those from high-performing sires (17.67 ± 1.09%). Conversely, the percent of TUNEL positive sperm cells in samples following gradient purification was significantly increased (*p* < 0.0001) in samples from high-performing sires (12.44 ± 0.95%) compared to those from low-performing sires (5.95 ± 0.71%).

### Morphology assessment via fluorescent microscopy

**Figure 2:**
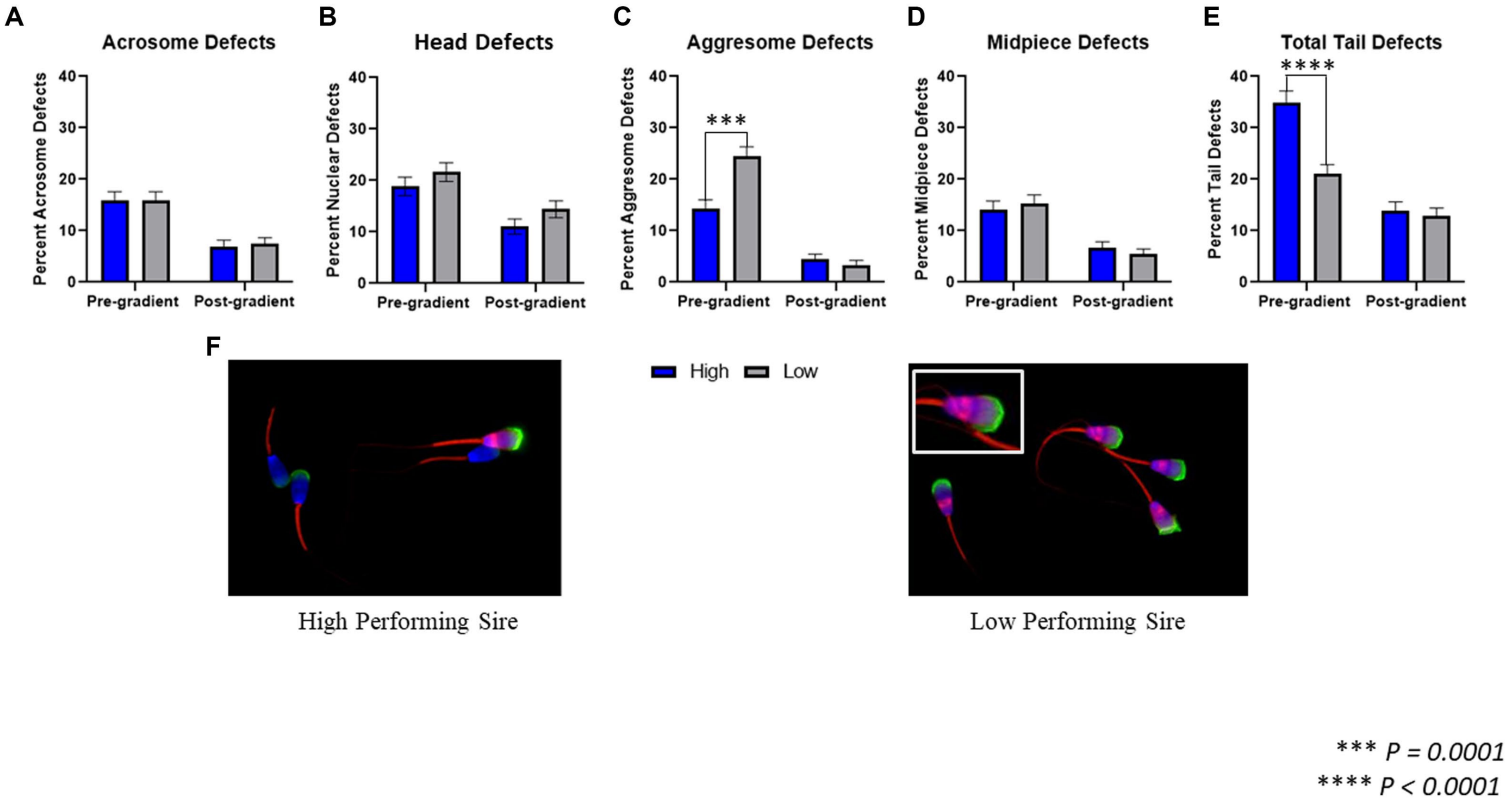
Morphological assessment fluorescent microscopy. **(A)** When comparing spermatozoa from high and low-performing sires both prior to gradient purification, termed pre-gradient samples, as well as post-gradient samples, there were no significant differences in the incidence of acrosomal defects (*p* = 0.978; *p* = 0.795). **(B)** In both pre-gradient and post-gradient samples, there were no differences in nuclear defects (*p* = 0.284; *p* = 0.132) between high and low-performing sires. **(C)** In pre-gradient samples, low-performing sires had a significantly higher incidence of aggresome defects in the head (*p* = 0.0001) compared to high-performing sires, however in post-gradient samples, there was no difference observed between high and low sires (*p* = 0.390). **(D)** In both pre-gradient and post-gradient samples, there were no differences in midpiece defects (*p =* 0.601; *p* = 0.401) between high and low-performing sires. **(E)** In pre-gradient samples, high-performing sires had a significantly higher incidence of tail defects (*p* < 0.0001) compared to low-performing sires, however in post-gradient samples, there was no difference observed between high and low sires (*p* = 0.623; *n* = ~450 cells per classification for all evaluations). **(F)** Representative images of fluorescently labeled spermatozoa from a high-performing sire (left) and a low-performing sire (right).

The following morphological regions were evaluated for the presence of defects and/or aggregated protein accumulation: acrosome, nucleus/head, midpiece, and tail principle piece (Figure 2).In pre-gradient samples, there was no difference observed in acrosomal defects (*p* = 0.978) between high-performing (15.88 ± 1.69%) and low-performing sires (15.94 ± 1.62%). There was also no difference in nuclear defects (*p* = 0.284) or midpiece defects (*p* = 0.601) between high-performing (18.88 ± 1.81%; 14.16 ± 1.62%) and low-performing sires (21.65 ± 1.83%; 15.35 ± 1.60%). In pre-gradient samples, low-performing sires had an increased incidence of aggresome defects in the head of the spermatozoa (*p* = 0.0001; 24.41 ± 1.91%) compared to high-performing sires (14.38 ± 1.63%). Alternatively, high-performing sires had increased total tail defects (*p* < 0.0001; 34.98 ± 2.20%) when compared to low-performing sires (21.06 ± 1.81%).

In post-gradient samples, there were no differences in acrosome defects (*p* = 0.795) or nuclear defects (*p* = 0.132) between high-performing (6.98 ± 1.21%; 11.04 ± 1.49%) and low-performing sires (7.43 ± 1.24%; 14.41 ± 1.67%). Similarly, there were no differences in aggresome defects (*p* = 0.390) or midpiece defects (*p* = 0.401) between high-performing (4.5 ± 0.98%; 6.76 ± 1.12%) and low-performing sires (3.38 ± 0.86%; 5.41 ± 1.07%). Finally, in post-gradient samples, there was also no difference in total tail defects (*p* = 0.623) between high-performing (13.96 ± 1.65%) and low-performing sires (12.84 ± 1.59%).

### Assessment by image-based flow cytometry

#### Fixed samples

**Figure 3:**
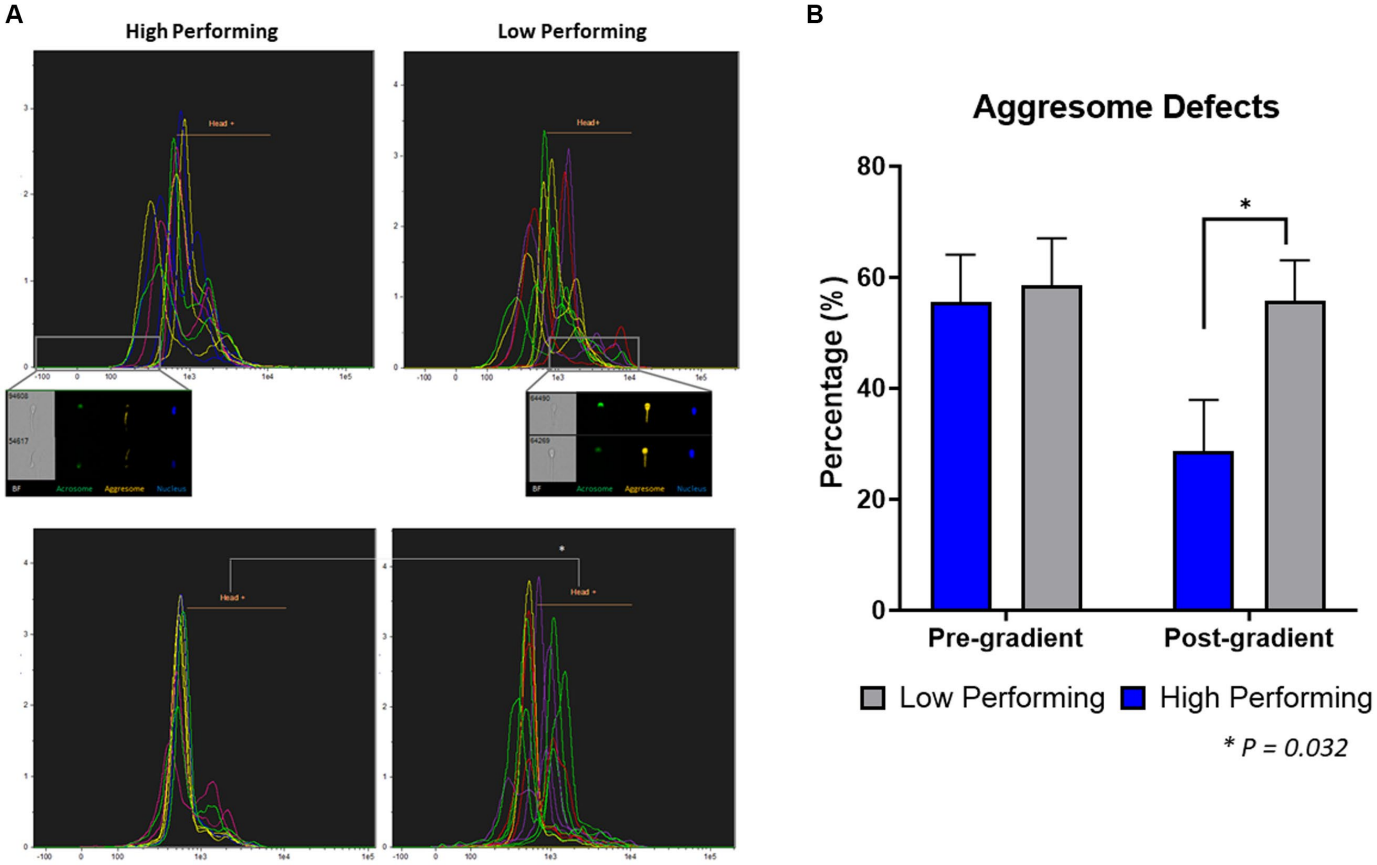
Quantification of aggresome defects in heads of spermatozoa. **(A)** In pre-gradient samples, there was no difference in the percent of spermatozoa with aggresome defects in the head (*p* = 0.818) between high and low-performing sires. **(B)** In post-gradient samples, low-performing sires had a significantly higher incidence of aggresome defects in the head (*p* = 0.03) compared to high-performing (*n* = ~90,000 cells per classification).

To quantify the aggresome content in spermatozoa from high and low-performing sires, fixed samples were stained and analyzed using image-based flow cytometry (Figure 3; Supplementary Figure S1). In samples prior to gradient purification, there was no difference (*p* = 0.818) between the aggresome content in the sperm heads from low-performing sires (58.53 ± 8.53%) compared to high-performing sires (55.53 ± 8.53%). Additionally, there was no difference (*p* = 0.132) between the total aggresome content in sperm cells from low-performing sires (94.42 ± 1.47%) compared to high-performing sires (97.71 ± 1.47%; Supplementary Figure S1A). In samples following gradient purification, sperm cells from low-performing sires had significantly increased incidence (*p* = 0.032) of aggresome content in their heads (55.78 ± 7.24%) compared to sperm cells from high-performing sires (28.68 ± 9.23%). Alternatively, there was again no difference (*p* = 0.217) between total aggresome content in sperm cells from low sires (90.4 ± 2.39%) compared to high sires (95.35 ± 3.05%; Supplementary Figure S1B), which indicates consistent labeling in the tail midpiece.

#### Live samples

**Figure 4:**
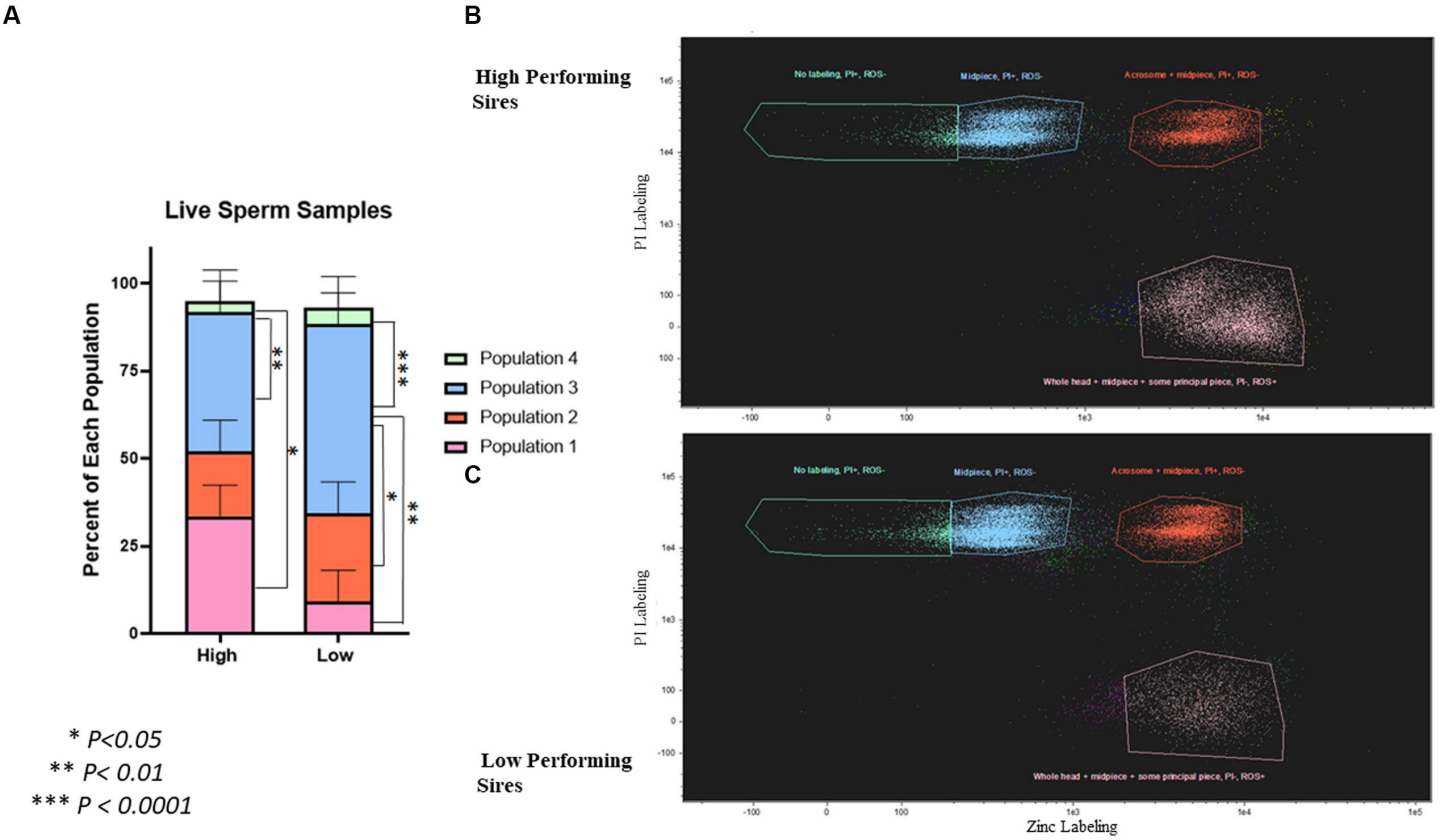
Quantification of spermatozoa viability and capacitation status in live, post-gradient samples. **(A)** Differences in populations of live, post-gradient samples (*n* = ~35,000 cells per classification) ranging from live, non-capacitated (Population 1) to moribund, post-capacitated spermatozoa (Population 4). Population 1 = zinc labeling in the whole head + midpiece + proximal principal piece, PI-, ROS+, Population 2 = zinc labeling in the acrosome + midpiece, PI+, ROS-, Population 3 = zinc labeling in the midpiece only, PI+, ROS-, and Population 4 = no zinc labeling, PI+, ROS-. In high-performing sires, there was a significantly higher percentage of Population 1 (*p =* 0.024), and Population 3 (*p* = 0.008) when compared to Population 4. In low-performing sires, there was a higher percentage of Population 3 that all others: Population 1 (*p* = 0.002), Population 2 (*p* = 0.032), and Population 4 (*p* = 0.0007). **(B)** Scatterplot showing the segregation of populations in high-performing sires. **(C)** Scatterplot showing the segregation of populations in low-performing sires. Segregation by ROS is shown in Supplementary Figure S2.

To evaluate the viability of live spermatozoa following gradient purification, sperm cells were co-stained with zinc ion, viability, and ROS probes; and analyzed using image-based flow cytometry (Figure 4; Supplementary Figure S2). The populations of spermatozoa in these samples segregated as follows: Population 1 (zinc labeling in the whole head + midpiece + some principal piece, PI-, ROS+), Population 2 (zinc labeling in the acrosome + midpiece, PI+, ROS-), Population 3 (zinc labeling in the midpiece only, PI+, ROS-), and Population 4 (no zinc labeling, PI+, ROS-). In the high-performing sires, there was a significantly higher percentage of population 1 (33.48 ± 8.91%; *p* = 0.024) and population 3 (39.63 ± 8.91%; *p* = 0.008) when compared to population 4 (3.17 ± 8.91%). In the low-performing sires, there was a significantly higher percentage of population 3 (53.95 ± 8.91%) than all others: population 1 (9.15 ± 8.91%; *p* = 0.002), population 2 (25.22 ± 8.91%; *p* = 0.032), and population 4 (4.7 ± 8.91%; *p* = 0.0007).

## Discussion

This study used imaging techniques to evaluate spermatozoa from high and low-performing bulls, both prior to and following gradient purification, to identify candidate biomarkers of sire fertility and to understand how the presence of those biomarkers may be impacted by gradient purification. It has been documented in many species that utilizing a density gradient combined with centrifugation will enrich the population of motile, morphologically normal sperm cells in a given sample (19, 26, 27). Although, this also reduces the number of total cells that remain following the process. Spermatozoa from pre-gradient samples of low-performing sires had increased DNA damage, however, in post-gradient samples, the inverse was observed, and high-performing sires had increased DNA damage. Although surprising, this change in the incidence of DNA damage between low and high-performing sires pre- and post-gradient could be attributed to gradient purification having the most substantial impact on removing spermatozoa with DNA damage when those cells were also compromised with other defects, or already dead (28, 29). Gradient purification is not known to selectively remove spermatozoa containing DNA damage alone. If a greater percentage of DNA-damaged spermatozoa from low-performing sires were dead upon thawing the semen straws, there would be a greater number of cells removed from the post-gradient population, which was observed in this study. This could explain why there was more DNA damage remaining in the post-gradient samples from high-performing sires, particularly if there is a discrete cohort of live, motile spermatozoa with DNA damage in bull semen (30).

It is unexpected to label aggresomes in the heads of viable spermatozoa, although consistent labeling in the head was seen in some samples during this study. In pre-gradient samples evaluated via fluorescent microscopy, the most prevalent defect identified in low-performing sires was the increased aggresome content in the head, specifically located in the post-acrosomal sheath region. Conversely, the most prevalent defects identified in the high-performing sires were in the tail, which are commonly removed with gradient purification because of their impaired motility (19, 27, 31). This may partially explain the discrepancy between a sire’s ability to produce greater numbers of embryos *in vitro*, following a gradient purification protocol, compared to their lower conception rates when used to service females *in vivo,* denoted by lower SCR values (6, 22).

When unfolded or misfolded proteins are not correctly refolded or efficiently degraded, it gives other proteins the opportunity to interact with them, sometimes leading to the formation of large protein aggregates, known as aggresomes (32). Aggresomes have the potential to form in any cell type, are associated with cell death, and have been linked to neurodegenerative diseases such as Alzheimer’s and Huntington’s disease, as well as systemic amyloidosis (32, 33). Once aggresomes form in a cell, they need to be cleared via autophagic degradation to limit further aggregate enlargement (32, 34). If degradation fails to occur efficiently, cellular proteostasis becomes perturbed—leading to cell damage and potentially to apoptosis.

A previous study has also identified increased protein aggregates in defective spermatozoa (25). Additionally, it has been shown that not all types of aggresomes are equally cleared via the autophagic pathway in neuroblastoma cells, indicating that there is selectivity to this relationship and that some cells may be more susceptible to slower degradation than others (35). Furthermore, dense protein aggregates being located in the post-acrosomal sheath region could potentially impair the release of the sperm-oocyte activating factor (SOAF), and/or the degradation of sperm cytoskeletal components therefore impacting oocyte activation or male pronuclear progress during early zygotic development (25).

The use of image-based flow cytometry (IBFC) to quantify aggresome content in the heads of spermatozoa allowed for a much larger sample size to be evaluated than manual assessment by fluorescent microscopy—approximately 90,000 cells per sire classification. In post-gradient samples, low-performing sires had an increased incidence of aggresome defects in the head compared to high-performing sires. The large sample size that was evaluated could explain the difference in results from the fluorescent microscopy assessment and indicate more robust results using flow cytometry. From the pre-gradient to the post-gradient samples, high-performing sires had a 30% reduction in cells containing this defect, indicating that many positive cells were removed during gradient purification. This resulted in less variation between post-gradient samples from the high-performing sires. Samples from low-performing sires had a mere 3% reduction in cells containing this defect from the pre to the post-gradient evaluation, indicating that this defect alone is less impacted by gradient purification.

In the flow cytometric evaluation of live spermatozoa following gradient purification, four distinct populations were identified in the samples, with Population 1 being the most viable and Population 4 being the least viable. The understanding of how zinc labeling indicated capacitation status in this study was based on previous literature by Kerns et al. (13), and allowed for differences in early vs. late capacitation to be distinguished from one another. Population 1 was composed of spermatozoa that had not begun capacitation associated with zinc efflux, had no plasma membrane alteration indicated by a lack of PI-labeling, and had a presence of ROS. Population 2 was composed of spermatozoa in early-stage capacitation, had membrane alteration (PI+), and had less ROS. Population 3 was composed of spermatozoa in late-stage capacitation, had membrane alteration, and had less ROS. Finally, Population 4 was composed of spermatozoa that were fully capacitated or dead, had membrane alteration, and had no ROS present. The high-performing sires had significantly more non-capacitated, viable sperm cells, in Population 1; than non-viable sperm cells, in Population 4. The largest proportion of sperm cells from low-performing sires were in Population 3 and were therefore less viable.

Previous literature has shown that sensitivity to cryopreservation can result in premature capacitation, as well as changes in mitochondrial Ca^2+^ and ROS of spermatozoa. These molecular changes lead to the activation of a large conductance channel referred to as the mitochondrial permeability transition pore (mPTP), causing the release of Ca^2+^, ROS, and ATP from the sperm cell (36–38). Additionally, the literature has shown that in human, equine, and bovine spermatozoa, the presence of ROS such as O_2_^−^ is necessary for tyrosine phosphorylation, capacitation, and hyperactivation to occur (39). It is possible that spermatozoa from low-performing sires may be more sensitive to the cryopreservation or post-thaw processes, therefor resulting in an alteration of viable populations. Early onset of capacitation is detrimental to spermatozoa fitness because it sets in motion the events that lead to acrosomal exocytosis, followed shortly thereafter by apoptosis. Therefore, the timing of capacitation is crucial to fertilization success.

Image based flow cytometry was chosen for these evaluations due to it is unique ability to accurately analyze tens of thousands of sperm cells for one or more features of interest in just minutes, significantly increasing the sample size and decreasing variability that exists in manual evaluations (11, 40). Furthermore, IBFC allows for the evaluation of individual cells, in contrast to more traditional methods such as polyacrylamide gel electrophoresis, mass spectrometry, and western blotting. Although these methods have been used for the identification of numerous sperm proteins and the states of those proteins such as phosphorylation, they are limited by reporting the average for all spermatozoa within a sample, and consequently lack the sensitivity to differentiate between individual cells (40). Evaluating average values for a heterogeneous population can result in biologically relevant data being overlooked. Therefore, this study, alongside others, promotes the use of IBFC to identify potential biomarkers of spermatozoa quality (10–13, 40).

Although previous studies from our group have shown that there was no difference between groups of sires with varied SCR values in their ability to effectively fertilize oocytes and reach the pronuclear stage (6), there were differences in developmental competence and cellular stress indicators observed in the subsequent embryos (22). If the number of spermatozoa from low-performing sires that can reach and fertilize an oocyte has been reduced due to gradient purification in addition to having a higher proportion of less viable cells, then the population that reaches the oocyte may be enriched for defects such as aggresomes located in their heads. Upon incorporation into the fertilized oocyte, aggresomes may overwhelm protein degradation machinery in the zygote—potentially leading to the upregulation in autophagic activity and ROS production that was observed in early embryos produced by low-performing sires (22) (Fallon et al., submitted). This indicates that it may prove to be beneficial to evaluate semen separately for AI use *in vivo* vs. *in vitro* embryo production. The identification of this phenomenon could explain the malfunction of one cellular mechanism regulated by sire during early embryo development and could serve as a biomarker of spermatozoan quality and sire fertility in the future.

## Data availability statement

The original contributions presented in the study are included in the article/Supplementary material, further inquiries can be directed to the corresponding author.

## Author contributions

LF: Conceptualization, Data curation, Formal analysis, Investigation, Methodology, Validation, Visualization, Writing – original draft, Writing – review & editing. ED-M: Formal analysis, Investigation, Methodology, Writing – review & editing. LH: Data curation, Formal analysis, Investigation, Methodology, Writing – review & editing. PS: Data curation, Resources, Supervision, Writing – review & editing. MZ: Data curation, Formal analysis, Investigation, Methodology, Writing – review & editing. TS: Conceptualization, Funding acquisition, Resources, Supervision, Writing – review & editing. MO: Conceptualization, Data curation, Funding acquisition, Investigation, Project administration, Resources, Supervision, Visualization, Writing – review & editing.

## References

[1] Ferrara F Daverio R Mazzini G Bonini P Banfi G. Automation of human sperm cell analysis by flow cytometry. Clin Chem. (1997) 43:801–7. doi: 10.1093/clinchem/43.5.801, PMID: 9166234

[2] Wiltbank MC Baez GM Garcia-Guerra A Toledo MZ Monteiro PLJ Melo LF. Pivotal periods for pregnancy loss during the first trimester of gestation in lactating dairy cows. Theriogenology. (2016) 86:239–53. doi: 10.1016/j.theriogenology.2016.04.037, PMID: 27238438

[3] Albuja C Ortiz O López C Hernández Cerón J. Economic impact of pregnancy loss in an intensive dairy farming system. Vet México OA. (2019) 6. doi: 10.22201/fmvz.24486760e.2019.1.572

[4] De Vries A. Economic value of pregnancy in dairy cattle. J Dairy Sci. (2006) 89:3876–85. doi: 10.3168/jds.S0022-0302(06)72430-4 16960063

[5] Franco G Reese S Poole R Rhinehart J Thompson K Cooke R. Sire contribution to pregnancy loss in different periods of embryonic and fetal development of beef cows. Theriogenology. (2020) 154:84–91. doi: 10.1016/j.theriogenology.2020.05.021, PMID: 32535394

[6] Ortega MS Moraes JGN Patterson DJ Smith MF Behura SK Poock S. Influences of sire conception rate on pregnancy establishment in dairy cattle. Biol Reprod. (2018) 99:1244–54. doi: 10.1093/biolre/ioy141, PMID: 29931362 PMC6299248

[7] Attia S Katila T Andersson M. The effect of sperm morphology and sire fertility on calving rate of Finnish Ayrshire AI bulls. Reprod Domest Anim. (2016) 51:54–8. doi: 10.1111/rda.12645, PMID: 26660630

[8] Harstine BR Utt MD DeJarnette JM. Review: integrating a semen quality control program and sire fertility at a large artificial insemination organization. Animal. (2018) 12:s63–74. doi: 10.1017/S1751731118000319 29467056

[9] Bar-Chama N Lamb DJ. EVALUATION OF SPERM FUNCTION: what is available in the modern andrology laboratory? Urol Clin N Am. (1994) 21:433–46. doi: 10.1016/S0094-0143(21)00618-2 8059499

[10] Da Costa R Redmann K Schlatt S. Simultaneous detection of sperm membrane integrity and DNA fragmentation by flow cytometry: a novel and rapid tool for sperm analysis. Andrology. (2021) 9:1254–63. doi: 10.1111/andr.13017 33830681

[11] Perticarari S Ricci G Granzotto M Boscolo R Pozzobon C Guarnieri S. A new multiparameter flow cytometric method for human semen analysis. Hum Reprod. (2007) 22:485–94. doi: 10.1093/humrep/del415, PMID: 17079246

[12] Zuidema D Kerns K Sutovsky P. An exploration of current and perspective semen analysis and sperm selection for livestock artificial insemination. Animals. (2021) 11:3563. doi: 10.3390/ani11123563, PMID: 34944339 PMC8698075

[13] Kerns K Zigo M Drobnis EZ Sutovsky M Sutovsky P. Zinc ion flux during mammalian sperm capacitation. Nat Commun. (2018) 9:2061. doi: 10.1038/s41467-018-04523-y, PMID: 29802294 PMC5970269

[14] Rajabi-Toustani R Akter QS Almadaly EA. Methodological improvement of FITC-PNA staining In: Senger P, editor. Pathways to Pregnancy and Parturition. Vol. 28. 2nd ed. Grubstake way, Redmond: Current Conceptions Inc. (2019).

[15] Sutovsky P Aarabi M Miranda-Vizuete A Oko R. Negative biomarker based male fertility evaluation: sperm phenotypes associated with molecular-level anomalies. Asian J Androl. (2015) 17:554–60. doi: 10.4103/1008-682X.153847, PMID: 25999356 PMC4492044

[16] Flesch FM Gadella BM. Dynamics of the mammalian sperm plasma membrane in the process of fertilization. Biochimica et Biophysica Acta (BBA)—reviews on. Biomembranes. (2000) 1469:197–235. doi: 10.1016/S0304-4157(00)00018-6, PMID: 11063883

[17] Cornwall GA von Horsten HH Whelly S. Cystatin-related Epididymal Spermatogenic aggregates in the epididymis. J Androl. (2011) 32:679–85. doi: 10.2164/jandrol.111.012963, PMID: 21764901 PMC3217266

[18] Sutovsky P Neuber E Schatten G. Ubiquitin-dependent sperm quality control mechanism recognizes spermatozoa with DNA defects as revealed by dual ubiquitin-TUNEL assay. Mol Reprod Dev. (2002) 61:406–13. doi: 10.1002/mrd.10101, PMID: 11835586

[19] Maxwell W Parrilla I Caballero I Garcia E Roca J Martinez E. Retained functional integrity of bull spermatozoa after double freezing and thawing using PureSperm® density gradient centrifugation: double freezing of bull spermatozoa. Reprod Domest Anim. (2007) 42:489–94. doi: 10.1111/j.1439-0531.2006.00811.x 17845604

[20] Kuhn MT Hutchison JL Norman HD. Modeling nuisance variables for prediction of service sire fertility. J Dairy Sci. (2008) 91:2823–35. doi: 10.3168/jds.2007-0946 18565940

[21] Norman HD Hutchison JL VanRaden PM. Evaluations for service-sire conception rate for heifer and cow inseminations with conventional and sexed semen. J Dairy Sci. (2011) 94:6135–42. doi: 10.3168/jds.2010-3875, PMID: 22118101

[22] Lockhart KN Drum JN Balboula AZ Spinka CM Spencer TE Ortega MS. Sire modulates developmental kinetics and transcriptome of the bovine embryo. Reproduction. (2023). doi: 10.1530/REP-23-0030 (In press). 37672361

[23] Fallon L Clark K Ortega MS. 56 paternal contributions to early embryonic stress affect development in the bovine. Reprod Fertil Dev. (2021) 34:263–4. doi: 10.1071/RDv34n2Ab56, PMID: 35231311

[24] Ortega MS Kurian JJ McKenna R Hansen PJ. Characteristics of candidate genes associated with embryonic development in the cow: evidence for a role for WBP1 in development to the blastocyst stage. PLoS One. (2017) 12:e0178041. doi: 10.1371/journal.pone.0178041, PMID: 28542629 PMC5436885

[25] Kennedy CE Krieger KB Sutovsky M Xu W Vargovič P Didion BA. Protein expression pattern of PAWP in bull spermatozoa is associated with sperm quality and fertility following artificial insemination: PROTEIN EXPRESSION PATTERN OF PAWP. Mol Reprod Dev. (2014) 81:436–49. doi: 10.1002/mrd.22309, PMID: 24488940

[26] Eberhardt M Prochowska S Duszewska AM Van Soom A Olech W Niżański W. The influence of Percoll® density gradient centrifugation before cryopreservation on the quality of frozen wisent (*Bison bonasus*) epididymal spermatozoa. BMC Vet Res. (2022) 18:305. doi: 10.1186/s12917-022-03408-z, PMID: 35945588 PMC9364487

[27] Phillips TC Dhaliwal GK Verstegen-Onclin KM Verstegen JP. Efficacy of four density gradient separation media to remove erythrocytes and nonviable sperm from canine semen. Theriogenology. (2012) 77:39–45. doi: 10.1016/j.theriogenology.2011.07.012, PMID: 21803408

[28] Ahlering P Sutovsky M Gliedt D Branson K Miranda Vizuete A Sutovsky P. Sperm content of TXNDC8 reflects sperm chromatin structure, pregnancy establishment, and incidence of multiple births after ART. Syst Biol Reprod Med. (2020) 66:311–21. doi: 10.1080/19396368.2020.1801889, PMID: 32851881

[29] Tomlinson MJ Moffatt O Manicardi GC Bizzaro D Afnan M Sakkas D. Interrelationships between seminal parameters and sperm nuclear DNA damage before and after density gradient centrifugation: implications for assisted conception. Hum Reprod. (2001) 16:2160–5. doi: 10.1093/humrep/16.10.2160, PMID: 11574509

[30] Dogan S Vargovic P Oliveira R Belser LE Kaya A Moura A. Sperm protamine-status correlates to the fertility of breeding Bulls1. Biol Reprod. (2015) 92:124255. doi: 10.1095/biolreprod.114.124255, PMID: 25673563

[31] Morrell J Johannisson A Dalin A-M Rodriguez-Martinez H. Morphology and chromatin integrity of stallion spermatozoa prepared by density gradient and single layer centrifugation through silica colloids. Reprod Domest Anim. (2009) 44:512–7. doi: 10.1111/j.1439-0531.2008.01265.x, PMID: 18992087

[32] Garcia-Mata R Gao Y-S Sztul E. Hassles with taking out the garbage: aggravating Aggresomes: Aggresomal pathway for protein degradation. Traffic. (2002) 3:388–96. doi: 10.1034/j.1600-0854.2002.30602.x, PMID: 12010457

[33] Kopito RR. Aggresomes, inclusion bodies and protein aggregation. Trends Cell Biol. (2000) 10:524–30. doi: 10.1016/S0962-8924(00)01852-3 11121744

[34] Driscoll JJ Chowdhury RD. Molecular crosstalk between the proteasome, aggresomes and autophagy: translational potential and clinical implications. Cancer Lett. (2012) 325:147–54. doi: 10.1016/j.canlet.2012.06.016, PMID: 22781397

[35] Wong ESP Tan JMM Soong W-E Hussein K Nukina N Dawson VL. Autophagy-mediated clearance of aggresomes is not a universal phenomenon. Hum Mol Genet. (2008) 17:2570–82. doi: 10.1093/hmg/ddn157, PMID: 18502787 PMC2722889

[36] Gualtieri R Kalthur G Barbato V Di Nardo M Adiga SK Talevi R. Mitochondrial dysfunction and oxidative stress caused by cryopreservation in reproductive cells. Antioxidants. (2021) 10:337. doi: 10.3390/antiox10030337, PMID: 33668300 PMC7996228

[37] Kim J-S He L Lemasters JJ. Mitochondrial permeability transition: a common pathway to necrosis and apoptosis. Biochem Biophys Res Commun. (2003) 304:463–70. doi: 10.1016/S0006-291X(03)00618-1, PMID: 12729580

[38] Zinovkin RA Zamyatnin AA. Mitochondria-targeted drugs. Curr Mol Pharmacol. (2019) 12:202–14. doi: 10.2174/1874467212666181127151059, PMID: 30479224 PMC6875871

[39] O’Flahtery C Scarlata E. The protection of mammalian spermatozoa against oxidative stress. Reproduction. (2022) 164:921–31. doi: 10.1530/REP-220200 37021966

[40] Ortega-Ferrusola C Gil M Rodríguez-Martínez H Anel L Peña F Martín-Muñoz P. Flow cytometry in Spermatology: a bright future ahead. Reprod Domest Anim. (2017) 52:921–31. doi: 10.1111/rda.13043, PMID: 28815751

