# Supplementary material for: The development of new biomarkers of spermatozoa quality in cattle
Source: Front Vet Sci. 2023 Oct 12;10:1258295. doi: 10.3389/fvets.2023.1258295 (PMC10601460; doi:10.3389/fvets.2023.1258295)
Supplement: Supplementary file 1 [file Data_Sheet_1.docx]

***Supplementary Materials***

**The Development of New Biomarkers of Spermatozoa Quality in Cattle**

**Lindsey Fallon^1^, Edgar Diaz-Miranda^1,2^, Lauren Hamilton^1^, Peter Sutovsky^1,3^, Michal Zigo^1^, M. Sofia Ortega^1*^**

^1^Division of Animal Sciences, University of Missouri, Columbia, MO, United States

^2^Department of Veterinary, Universidade Federal de Viçosa, Viçosa, MG, Brasil

^3^Departments of Obstetrics, Gynecology & Women’s Health, University of Missouri, Columbia, MO, United States

***Correspondence:**

M. Sofia Ortega

1675 Observatory Drive | 758 | Madison, WI 53706

(608) 264- 1350

[sofia.ortega@wisc.edu](mailto:sofia.ortega@wisc.edu)

**
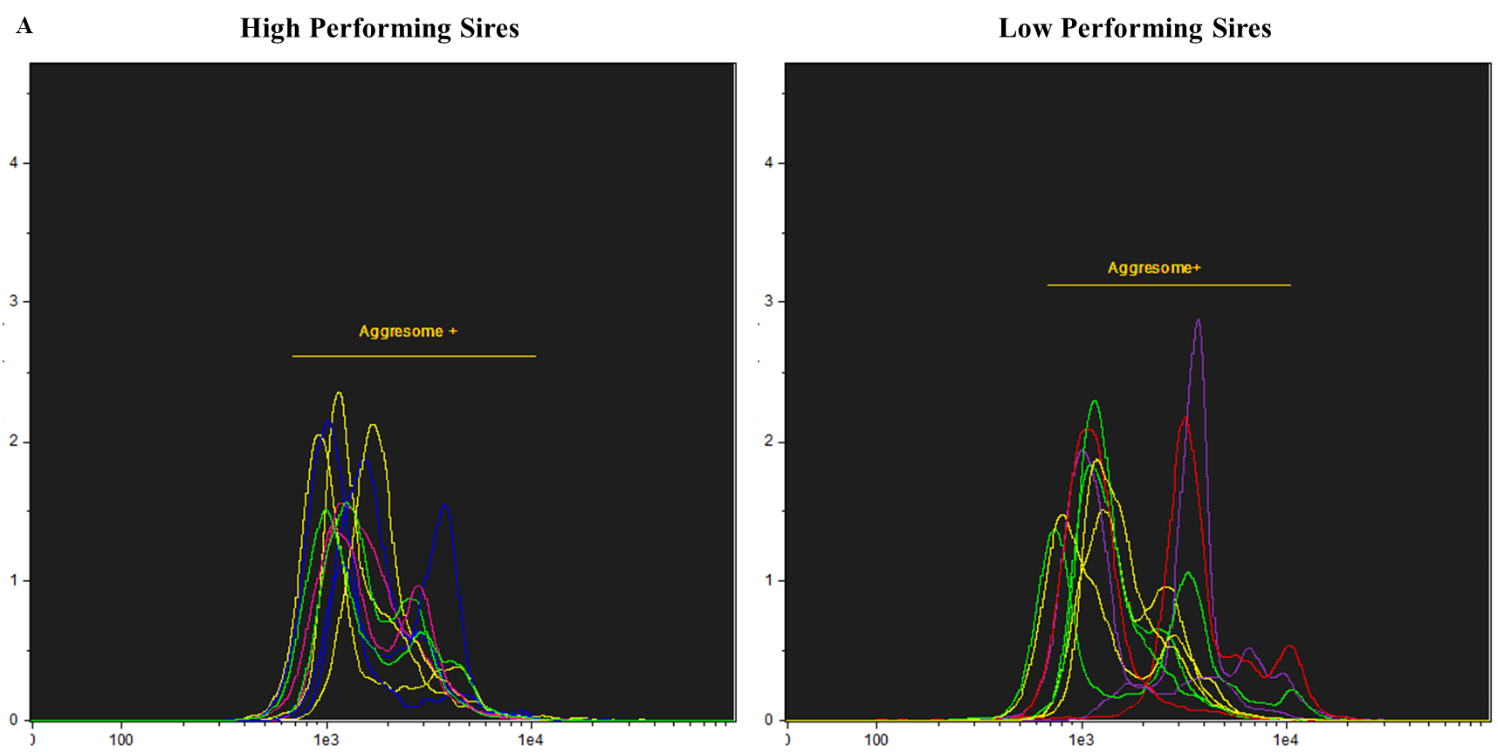
**

**
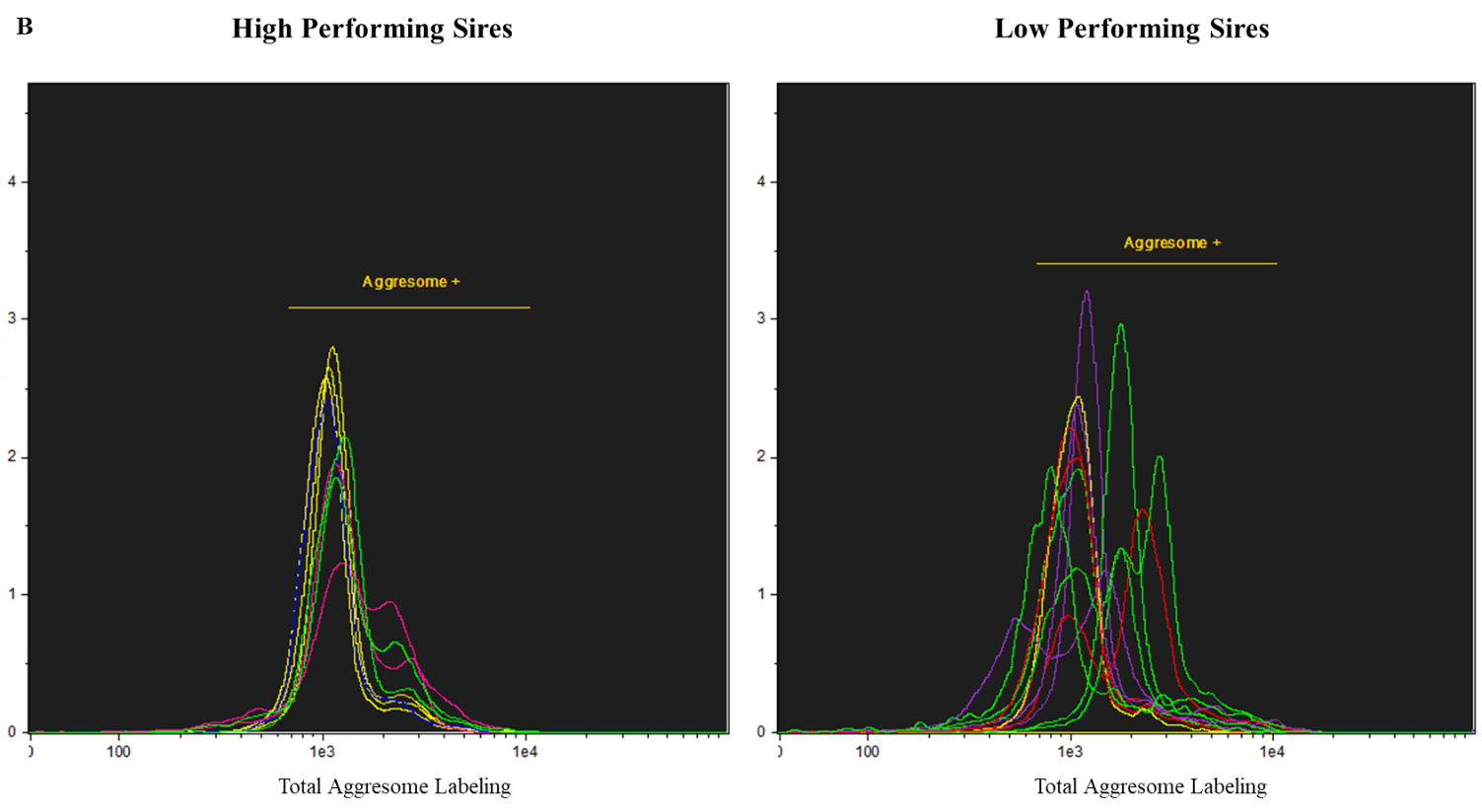
**

**Figure S1. Total sperm aggresome content.** (A) In pre-gradient samples, there was no difference in total aggresome content (*P*=0.132) of high and low performing sires. (B) In post-gradient samples, there was no difference in total aggresome content (*P*=0.217) of high and low performing sires, most likely due to signal contributed by tail midpiece.

**
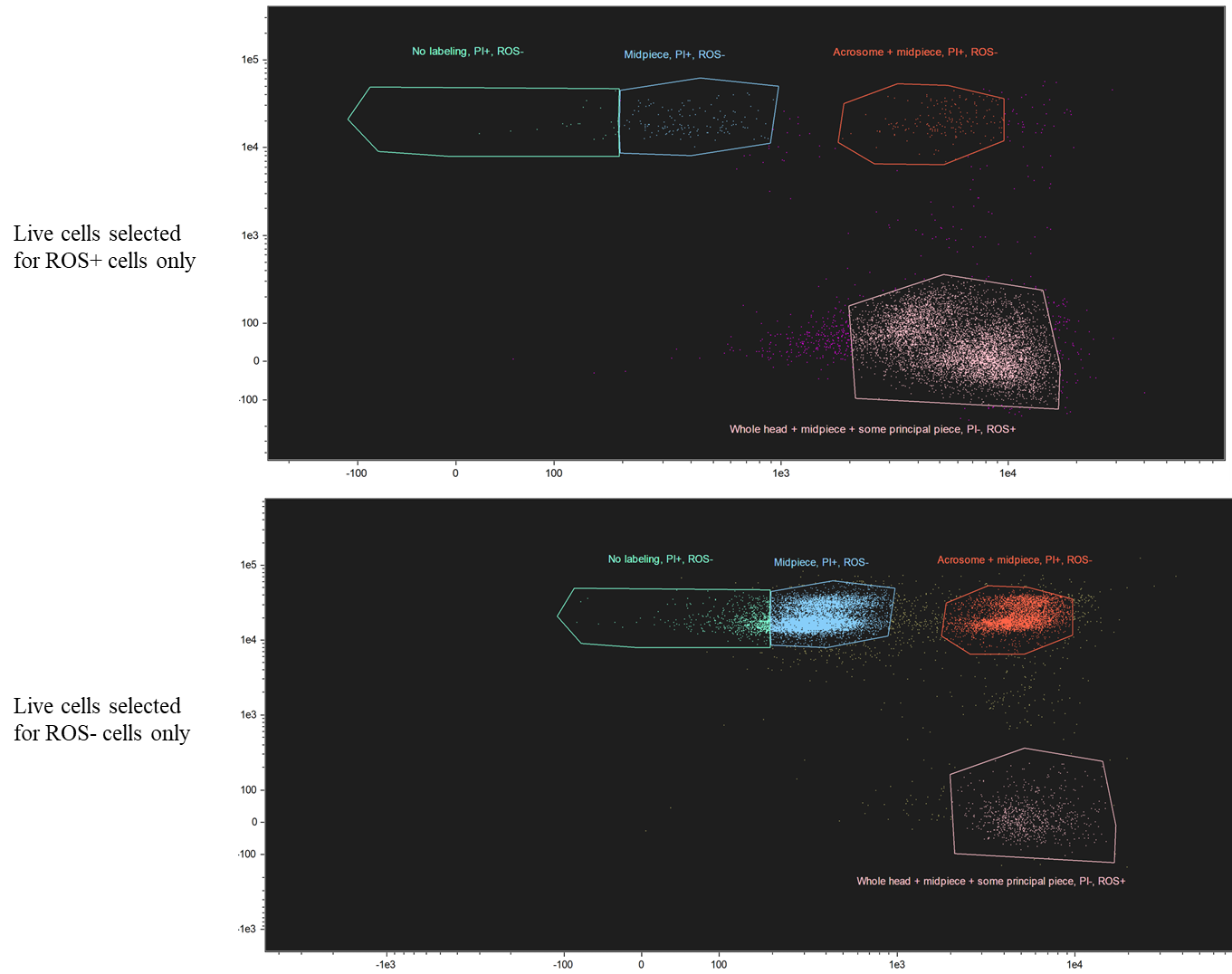
**

**Figure S2. Presence of ROS in live, post gradient samples.** (A) Differences in the presence of ROS in populations of live, post-gradient samples (n= ~35,000 cells per classification) ranging from a strong ROS signal in the midpiece to absence of ROS signal in the midpiece.
